# Effectiveness of a fetal magnetic resonance imaging scoring system for predicting the prognosis of pernicious placenta previa: A retrospective study

**DOI:** 10.3389/fphys.2022.921273

**Published:** 2022-08-11

**Authors:** Lue Zou, Pengyuan Wang, Zixuan Song, Xiaoxue Wang, Xueting Chen, Mingjie Zhang, Dandan Zhang

**Affiliations:** ^1^ Department of Radiology, Shengjing Hospital of China Medical University, Shenyang, China; ^2^ Department of Obstetrics and Gynecology, Shengjing Hospital of China Medical University, Shenyang, China; ^3^ Department of Health Management, Shengjing Hospital of China Medical University, Shenyang, China; ^4^ Department of Surgery, Shengjing Hospital of China Medical University, Shenyang, China

**Keywords:** pernicious placenta previa, postpartum hemorrhage, specificity, sensitivity, youden indexes, area under the curve

## Abstract

**Background:** The value of multiple magnetic resonance imaging (MRI) signs in predicting pernicious placenta previa (PPP) with placenta accreta spectrum disorders (PAS) is still controversial. This study aimed to investigate the value of a self-made fetal magnetic resonance imaging scoring system in predicting the different types of PAS in pernicious placenta previa and its associated risk of bleeding.

**Methods:** This retrospective study included 193 patients diagnosed with PPP based on MRI findings before delivery. Based on pathological and intraoperative findings, we divided patients into four groups: non-PAS, placental adhesion, placental implantation, and placenta percreta. Receiver operator characteristic curves of the MRI total score and placental implantation type were drawn using pROC packages in the R Studio environment, and cutoff values of each type were calculated, as well as diagnostic evaluation indexes, such as sensitivity, specificity, and the Youden index. Hemorrhage during surgery was compared between the groups.

**Results:** The boundary value between the non-PAS and placental adhesion was 5.5, that between placental adhesion and placental implantation was 11.5, and that between placental implantation and placenta percreta was 15.5 points. The respective specificities were 0.700, 0.869, and 0.958, and the respective sensitivities were 0.994, 0.802, and 0.577. The Youden indices were 0.694, 0.671, and 0.535, respectively. The median (minimum, maximum) quantities of hemorrhage during the operation in the non-PAS, placental adhesion, placental implantation, and placenta percreta groups were 225 (100, 3700), 600 (200, 6000), 1500 (300, 7000), and 3000 (400, 6300) ml, respectively. Hemorrhage was significantly different between the four groups (*p* < 0.001).

**Conclusion:** These results suggest that the proposed MRI scoring system could be an effective diagnostic tool for assessing PPP types and predicting the associated bleeding risk.

## Background

In a normal pregnancy, the placenta is attached to the uterine floor, anterior wall, posterior wall, or lateral wall. After 28 weeks of gestation, the placenta is still attached to the lower segment of the uterus, the lower margin reaches or exceeds the internal cervical os, or the position is lower than the fetal exposure, which is called placenta previa. Pernicious placenta previa (PPP) is clinically defined when the pregnancy is placenta previa and the placenta covers the scar of the original cesarean section. Since 2016, with the liberalization of the family planning policy in China, the incidence of PPP has almost shown a rapid growth trend with an increase in high-risk factors such as advanced pregnancy, the cesarean section rate, induced abortion rate, and history of uterine operation, which has increased by approximately 10 times (Kayem et al., 2013; Silver et al., 2015). PPP has gradually replaced postpartum uterine contraction weakness and postpartum hemorrhage, and become the first reason for emergency hysterectomy, which is another major problem of postpartum hemorrhage in obstetrics clinic that seriously affects the outcome of mothers and children and even threatens their safety (Bao et al., 2016).

Approximately half of women with PPP have placental implantation (PI) (Yu Lin and Yang, 2016), resulting in severe delivery complications. In 2018, the International Federation of Gynecology and Obstetrics (FIGO) collectively referred to different types of placental implant disorders such as placenta accreta spectrum disorders (PAS) (Jauniaux et al., 2018a). According to the invasion depth of placental villi, PAS can be divided into the following three categories: placental adhesion (PA), which only affects the superficial uterine muscle; PI, which involves the deep muscle layer of the uterus; and placenta percreta (PP), which penetrates the myometrium into the serous layer of the uterus and penetrates the serous layer into the adjacent organs and tissues of the uterus, such as the bladder, rectum, and pelvic wall (Jauniaux et al., 2018a). In April 2021, the *American Journal of Obstetrics and Gynecology* published a retrospective observational study that examined trends, risk factors, and perioperative outcomes of PAS (Matsuzaki et al., 2021). The incidence of PAS was 0.29% (8030/2 727 477 cases), and the incidences of PA, PI, and PP were 0.23, 0.03, and 0.04%, respectively. The incidence of PAS increased by 2.1% per quarter, from 0.27% in the fourth quarter of 2015 to 0.32% in the fourth quarter of 2017 (*p* = 0.004). Compared to women with non-PAS, those with PAS and a history of cesarean section had a higher risk of surgical complications [78.3% versus (vs.) 10.6%], severe maternal complications as defined by the Centers for Disease Control and Prevention (60.3 vs. 3.1%), bleeding (54.1 vs. 3.9%), clotting-related diseases (5.3 vs. 0.3%), shock (5.0 vs. 0.1%), urinary tract injury (8.3 vs. 0.2%), and death (0.25 vs. 0.01%) (all, *p* < 0.05). PPP patients with PAS are more likely to have premature delivery, perinatal hemorrhage, and other life-threatening complications. In clinical practice, it is necessary to choose an appropriate treatment plan according to the location of the placenta, implantation site, and degree of PPP. Therefore, prenatal evaluation of PPP with implantation and the implantation type is extremely important.

Currently, imaging is the main method used for the preoperative diagnosis of PPP in clinical practice. Ultrasonography (US) and magnetic resonance imaging (MRI) have been widely used. Among them, US has the advantages of safety, economy, speed, and convenience and is the most widely used diagnostic imaging method in clinical practice. The characteristics of the images are as follows: disappearance of the posterior placental space, formation of lacunae in the placenta or thickening of the placenta, rich blood flow behind the placenta or even the vortex, and roughness of the uterine bladder wall (Bowman et al., 2014). However, owing to its strong subjectivity, poor repeatability, and ease of being affected by abdominal wall thickness, intestinal gas, and other shortcomings, the application of US is limited. Compared with ultrasound, MRI has the advantages of repeatability and multidirectional and multiparameter imaging, large field of view, high soft tissue contrast and spatial resolution and is independent of the effect of intestinal gas and abdominal wall thickness (Baughman et al., 2008; Torrents-Barrena et al., 2019). The characteristics of the images are as follows: the gap between the placenta and uterus disappears, abnormal signals or continuity of the myometrium of the uterus are interrupted, and signals of the myometrium, bladder wall, and mucosa are discontinuous (Leyendecker et al., 2012).

In China, the average spending of MRI is higher than that of US (Fan et al., 2020). However, a large number of studies have shown that MRI has advantages in diagnosing placenta implantation depth, implantation scope, and invasion of bladder and parauterine. It often can be used as an important basis of optimizing cesarean section to decrease intraoperative bleeding and reduce the uterus resection rate. In particular, T2-weighted imaging (T2WI) of the low signal zone in the placenta, blood vessels in placental hyperplasia, and uterine exudation have good predictive value in the prenatal diagnosis of PAS (sensitivity, 82.2–100%; specificity, 84.0–100%) (D'Antonio et al., 2014; Meng et al., 2013). Whether the combination of multiple MRI signs can be used to predict PPP with PAS has not been clarified yet. Therefore, in this study, an MRI signal-based scoring model was constructed by screening and integrating the MRI signs of patients with PPP in the middle and late stages of pregnancy, and its value in PPP with PAS and adverse clinical outcomes was investigated.

## Methods

### Ethics statements

The study was approved by the ethics committee of Shengjing Hospital (approval number: 2022PS132K). All patients were informed of the relevant matters before study initiation. The patients voluntarily entered the study and signed an informed consent form.

### Study design and patients

**FIGURE 1:**
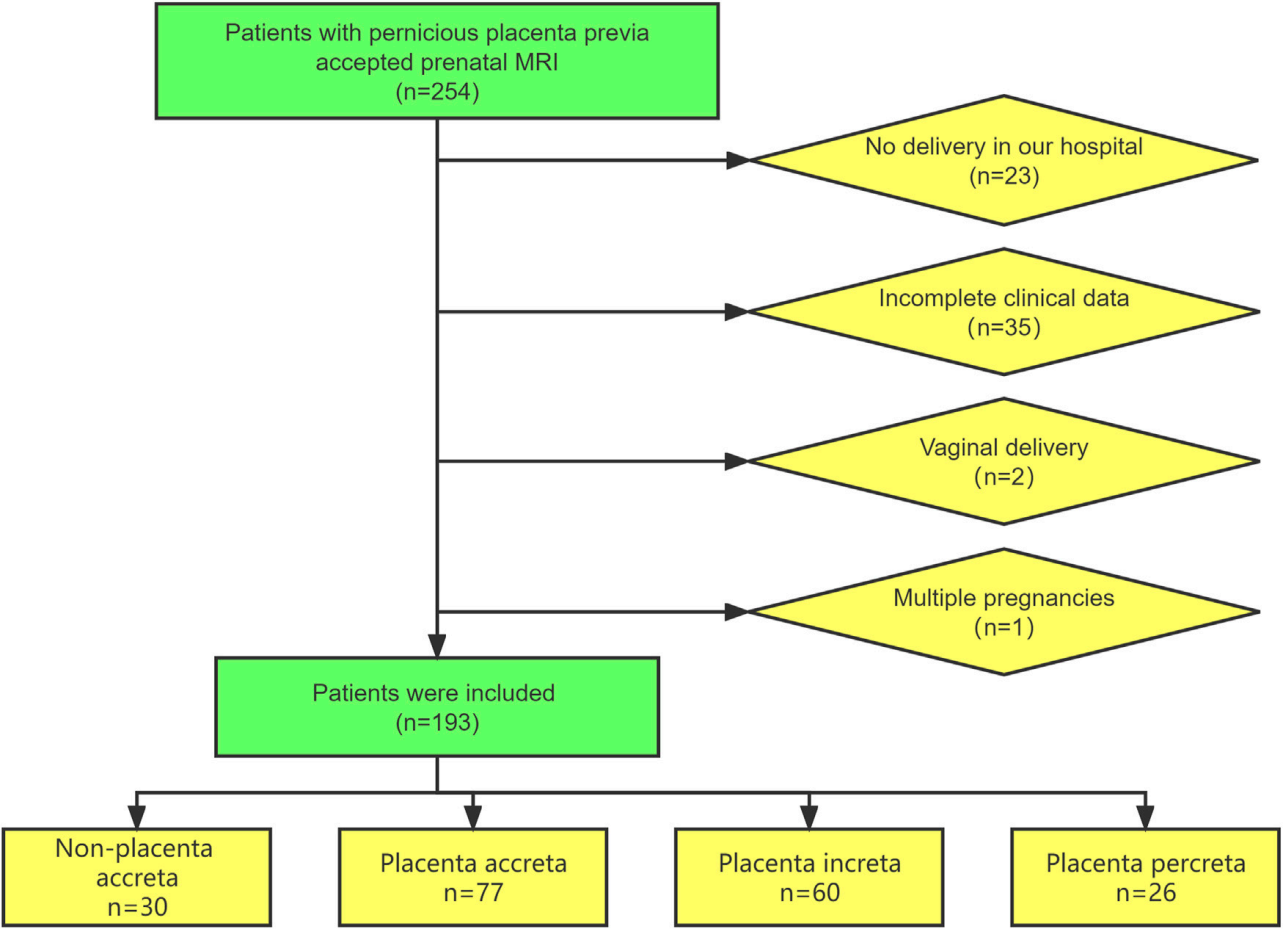
The flowchart of patient selection.

This retrospective study was conducted on patients with suspected PPP who underwent prenatal MRI examinations at Shengjing Hospital of China Medical University from January 2018 to December 2021. The inclusion criteria were as follows: 1) history of cesarean section and 2) women at 28–39 gestational weeks. The exclusion criteria were as follows: 1) not delivery at our hospital, 2) vaginal delivery, 3) multiple pregnancies, and 4) incomplete clinical data. The patient inclusion process is illustrated in Figure 1.

### Data collection

Clinical data and MRI findings at 28–39 weeks of gestation were collected. Clinical data included patient age, gestational age, newborn weight, and blood loss during the cesarean section. The amount of blood loss was measured using gravimetric, visual, shock index, and estimated blood loss methods.

The Gyroscan Intera 1.5T MRI system (Philips) was used to perform coronal pelvic scanning with the patient in either the supine or lateral decubitus position, followed by axial, sagittal, and coronal placental scanning based on the fetal position. The gradient strength of the magnet was 40 mT/m with a slew rate of 150 mT/m/s, and the slice thickness was 4.5–5.0 mm, the slice gap was 1–1.5 mm with matrix 256 × 256. Using Sense technology, turbo field echo sequences were used in T1-weighted imaging [repetition time (TR), 10 ms; echo time (TE), 4.6 ms]; high-resolution Sense single-shot turbo spin echo (sSSh-TSE) sequences were used for T2WI (TR, 635–640 ms; TE, 150 ms); SPAIR (SPectral Attenuated Inversion Recovery) was used for fat suppression. The following types of PAS were determined by a comprehensive diagnosis combined with surgery and pathology. 1) Non-PAS: The placenta can be delivered spontaneously. 2) PA: The pathological examination shows abnormal adherence of the placenta to the myometrium and absence of the bottom decidua. During the operation, the placenta remains in the uterine cavity and must be manually removed. Bleeding may occur on the surface of the placenta. 3) PI: Pathological findings show that the villi have invaded the myometrium. During the operation, the implanted part cannot be removed by itself, and it is difficult to remove manually, with a lot of bleeding. 4) PP: In pathological sections, the placental villi have invaded the serosal layer of the uterus and sometimes they will invade the adjacent pelvic organs. During surgery, the placenta can be directly observed to break through the serosal layer of the uterus. The placenta cannot be manually detached, and uterine rupture and massive bleeding can occur.

### Scoring system

Image reading was performed using the Neusoft PACS workstation (version 5.5.6). Radiographs were reviewed independently by two specialist prenatal diagnostic radiologists (10, and 8 years experienced) blinded to outcome and scored without knowing the surgical and pathological results. When there was a disagreement, the chief radiologists (20 years experienced) participated in the discussion, and a consensus was reached. The PPP scoring system (Table 1) was based on the FIGO guidelines (Jauniaux et al., 2018b) and the joint consensus on PAS published by the Society of Abdominal Radiology and European Society of Urogenital Radiology in 2020 (Jha et al., 2020). A total of 10 MRI signs and one clinical feature were selected, including number of previous cesarean deliveries, placental location, placental/uterine bulges, placental heterogeneity, T2-dark bands in placenta, abnormal intraplacental vascularity, abnormal vascularization of the placental bed, loss of T2 hypointense interface, bladder wall interruption, penetrating PI, and myometrial thinning and interruption. The system consists of 11 scoring items that are divided into 0, 1, and 2 points according to severity. The total score reflects the severity of PPP.

**TABLE 1:** Magnetic resonance imaging-based scoring system for pernicious placenta previa.

MR characteristics	Sequences	0	1	2
Placenta position	T2WI	Normal	Marginal placental previa or low lying placenta	Completely placental previa
Placental/uterine bulge	T2WI	Normal	Suspected	Yes
Placental heterogeneity	T2WI	None	Suspected	Yes
T2-dark bands in placenta	T2WI	None	1 place	>=2 places
Abnormal intraplacental vascularity	T2WI	None	1 place	>=2 places
Abnormal vascularization of the placental bed	T2WI	None	1 place	>=2 places
Loss of T2 hypointense interface	T2WI	Normal	Suspected	Yes
Bladder wall interruption	T2WI	Normal	Blurring	Interruption
Penetrating placenta implantation	T2WI	None	Suspected	Yes
Myometrial thinning and interruption	T1WI&T2WI	Normal	Thickness≤1 mm	Interruption
Number of previous cesarean deliveries	-	-	1	>=2

### Statistical analysis

The measurement data conforming to a normal distribution are represented as ( $\bar{\text{X}}$ ± s). Measurement data with a non-normal distribution are expressed as median (minimum, maximum). Univariate analysis of variance was used to compare the means of multiple groups. The rank-sum test was used to compare MRI total score and intraoperative blood loss between the four groups. Receiver operating characteristic (ROC) curves of the MRI total score and placenta accreta type were drawn using R package pROC, the boundary value of each type was calculated, and diagnostic evaluation indexes such as sensitivity, specificity, and the Youden index were calculated.

Data were analyzed in an R Studio environment using R software (version 3.6.3; R Foundation for Statistical Computing, Vienna, Austria; http://www.r-project.org). Statistical significance was set at *p* < 0.05.

## Results

### Patient characteristics

In total, 193 patients with suspected pernicious placenta previa were enrolled in this study. All patients underwent a complete prenatal MRI examination. Each patient was scored according to the scoring system shown in Table 1, and the clinical data were collected. The general characteristics and MRI scores of the patients are shown in Tables 2, 3, respectively. MRI total scores of non-placenta accreta, placenta accreta, placenta increta, and placenta percreta showed significant differences (*p* < 0.001).

**TABLE 2:** Patient general characteristics.

	Non-placenta accreta	Placenta accreta	Placenta increta	Placenta percreta	*p* value
Numbers	30	77	60	26	
Age	31.9 ± 3.8	32.8 ± 4.4	34.1 ± 4.2	32.8 ± 3.8	0.108^▲^
Cesarean sections history					0.024*△
Once	29	64	43	20	
Twice or more	1	13	17	6	
Placenta previa type					0.013*△
Completely previa	24	57	46	26	
Low placenta	6	20	14	0	
Childbirth week	36.7 (29.3–37.9)	36.6 (28.4–38.7)	36.9 (28.1–38.9)	36.8 (29.0–38.9)	0.297●
Neonatal weight (g)	2765.1 ± 559.0	2744.8 ± 570.9	2867.2 ± 646.6	2976.8 ± 689.5	0.099^▲^

*: *p*<0.05; ▲: One-way ANOVA; △: Fisher’s precision probability test; ●: Kruskal-Wallis test.

**TABLE 3:** Four groups of patients with their corresponding magnetic resonance imaging scores.

	Non-placenta accreta	Placenta accreta	Placenta increta	Placenta percreta
Numbers	30	77	60	26
Average score	5.6	9.4	12.8	15.5
Standard deviation	2.5	2.9	2.5	3.5
Minimum	3	5	6	9
Maximum	13	19	20	21
95% CI lower limit	0.8	3.7	8.0	8.7
95% CI upper limit	10.5	15.1	17.6	22.3

CI, confidence interval.

**FIGURE 2:**
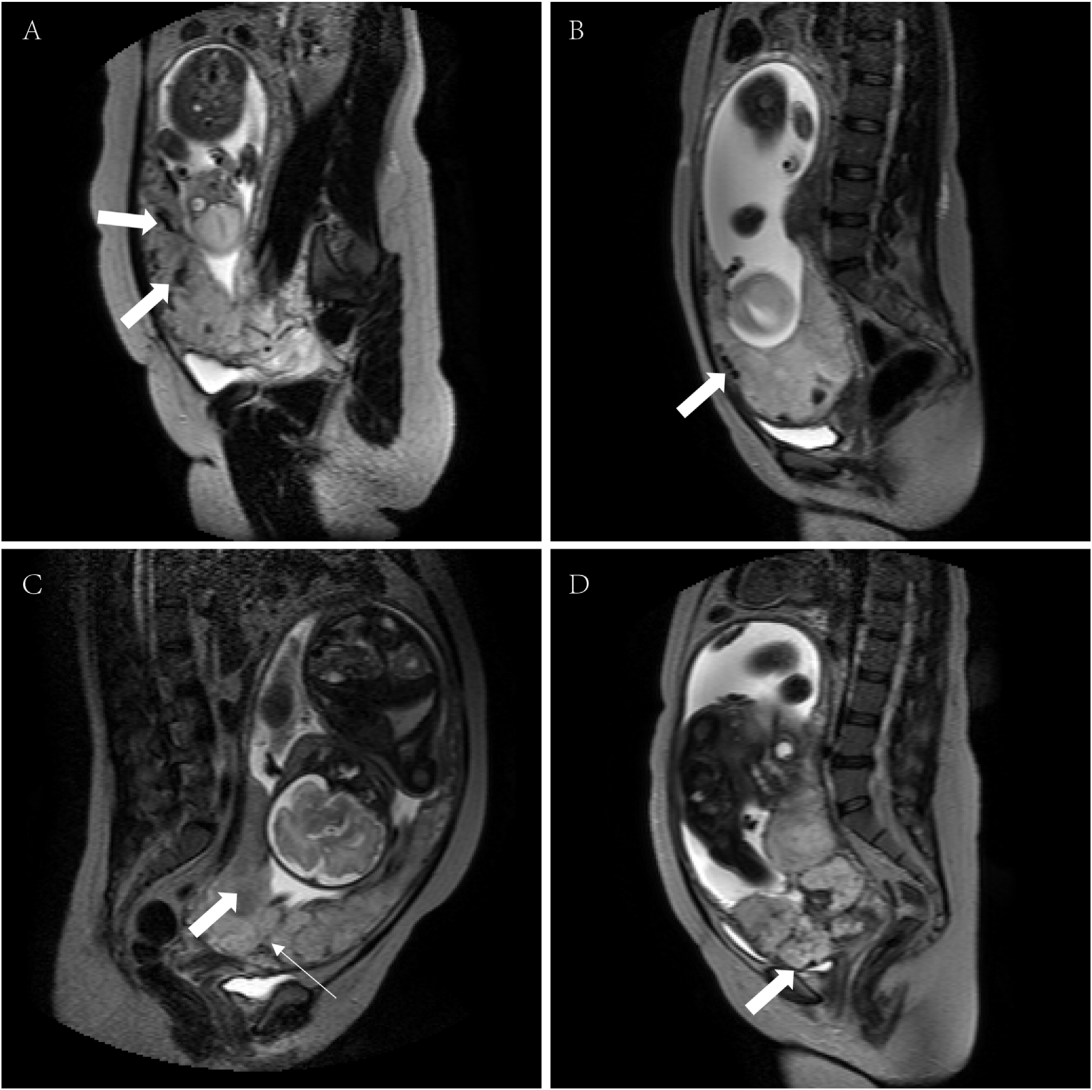
MRI signs associated with PPP (T2WI and sSSh-TSE sequences). **(A)** Intraplacental abnormal vascularity (arrows), **(B)** abnormal vascularization of the placental bed (arrow); **(C)** heterogeneous placenta (broad arrow) with a dark band on T2WI (narrow arrow); and **(D)** serosal surface of the bladder disruption (arrow). MRI, magnetic resonance imaging; PPP, pernicious placenta previa; T2WI, T2-weighted imaging; sSSh-TSE, Sense single-shot turbo spin echo.

Some typical MRI images are shown in Figures 2A–D. Among the peculiar features of the MRIs, there is an abnormal intraplacental vascularity, that can be deduced from tortuous enlarged flow voids on T2-weighted images deep within the placenta. Furthermore, a diagrammatic representation abnormal vascularization of the placental bed is demonstrated by prominent vessels in the placental bed with disruption of the uteroplacental interface, and the vessels may be accompanied by extensive neovascularization around the bladder, uterus, and vagina. Heterogeneous placenta is represented on the diagram as altered background parenchymal signal, which is in addition to the T2-dark bands and abnormal intraplacental vascularity. When the bladder wall is interrupted, there is irregularity and disruption of the normal hypointense bladder wall, with placental tissue protruding into the bladder lumen, usually accompanied by “bladder vessel signs.”

### Receiver operating characteristic curve predicted the boundary value of pernicious placenta previa according to the magnetic resonance imaging evaluation

**FIGURE 3:**
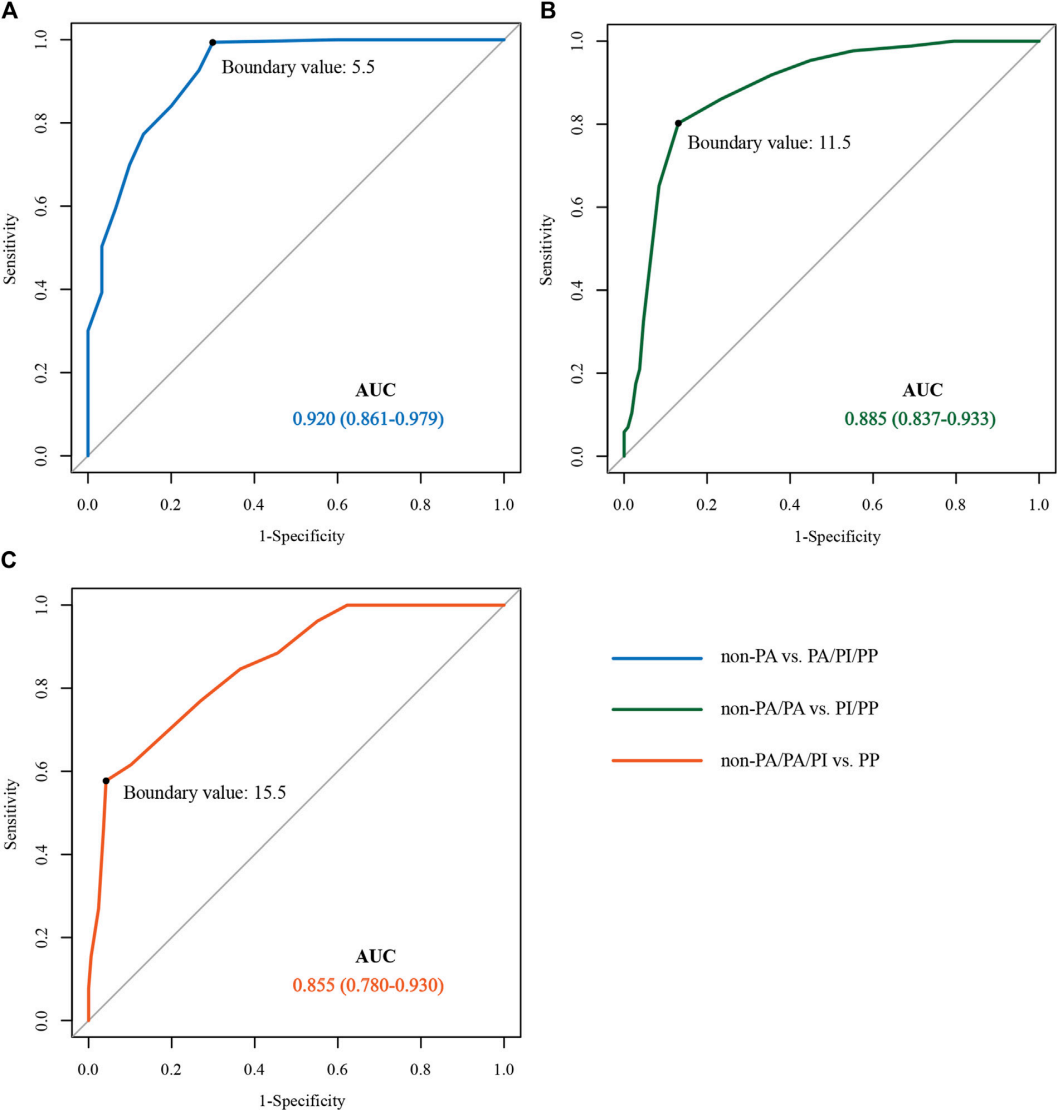
ROC curve for predicting different types of pernicious placenta previa based on the MRI scoring system. **(A)** non-PA vs. PA/PI/PP ROC curves; **(B)** non-PA/PA vs. PI/PP ROC curves; **(C)** non-PA/PA/PI vs. PP ROC curves. ROC, receiver operating characteristic; MRI, magnetic resonance imaging; PA, placental adhesion; PI, placental implantation; PP, placenta percreta.

By drawing ROC curves, the MRI evaluation boundary values for the different types of PAS were calculated (Figures 3A–C). A value of 0.920 was found for the area under the curve when the boundary value of non-PA vs. PA/PI/PP was 5.5, and the sensitivity and specificity were 99.40 and 70.00%. A value of approximately 0.885 was recorded for area under the curve when the boundary values of Non-PA/PA vs. PI/PP was space 11.5, and the sensitivity and specificity were 80.21 and 86.94%, and the area under the curve was 0.855 when the boundary values of Non-PA/PA/PI vs. PP was 15.5. The Youden indices were 0.694, 0.671, and 0.535, respectively. The prediction accuracies for each group are presented in Table 4.

**TABLE 4:** Pathological predictions in the four groups of patients.

	Compare A	Compare B	Compare C
AUC(95% CI)	0.920 (0.861–0.9785)	0.885 (0.837–0.9331)	0.855 (0.7796–0.9304)
Boundary value	5.5	11.5	15.5
Specificity	0.700	0.869	0.958
Sensitivity	0.994	0.802	0.577
Positive predict value	0.947	0.831	0.682
Negative predict value	0.955	0.845	0.936
Youden index	0.694	0.671	0.535
Positive likelihood ratio	3.313	6.132	13.764
Negative likelihood ratio	0.009	0.227	0.442
Kappa value	0.780	0.670	0.570

Compare A: Non-placenta accreta vs. placenta accreta/placenta increta/placenta percreta.

Compare B: Non-placenta accreta/placenta accreta vs. placenta increta/placenta percreta.

Compare C: Non-placenta accreta/placenta accreta/placenta increta vs. placenta percreta.

### Postpartum blood loss in each group

**FIGURE 4:**
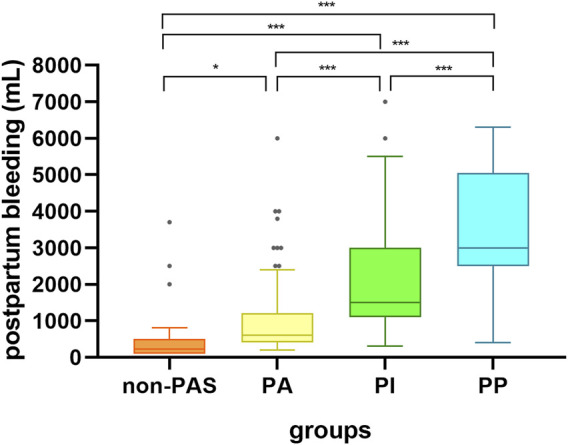
Box diagram representation of bleeding volume in the four groups of patients. non-PAS: no placenta accreta spectrum disorders; PA, placental adhesion; PI, placental implantation; PP, placenta percreta; *p < 0.05; ***p < 0.001.

The rank-sum test was used to compare the postpartum bleeding volumes of the non-PAS, PA, PI, and PP groups, and the results showed that the median numerical bleeding volumes were 225 (100, 3700), 600 (200, 6000), 1500 (300, 7000), and 3000 (400, 6300) ml, respectively. There was a significant difference in blood loss between the groups (all, *p* < 0.05). The amounts of blood loss in the four groups are shown in Figure 4.

## Discussion

This study found that the scoring model based on MRI signs was valuable in the diagnosis of PPP with or without PAS and prediction of adverse clinical outcomes. Based on ultrasound diagnosis before delivery, placental MRI can be performed in the middle-late stages of pregnancy to evaluate the risk of PI and adverse clinical outcomes, which is beneficial for the refined and stratified management of patients during pregnancy.

The value of MRI in PAS diagnosis has received increasing attention recently (Srisajjakul et al., 2021). Few studies have been conducted on the MRI diagnosis of PPP. It is important to establish a diagnostic scoring model for PAS to quantify MRI signs and improve diagnostic value (D'Antonio et al., 2014; Ishibashi et al., 2020). Ueno et al. (2016) developed the first MRI-based predictive model for placenta implantation in 2016. A Likert scale was used to score six MRI signs, including dark banding on T2-weighted images, intraplacental abnormal vascularity, placental bulge, heterogeneous placenta, myometrial thinning, and placental protrusion sign, on a scale of 1–5, and the total score was summed. Statistical comparison showed that the MRI scoring system had good diagnostic efficacy for PAS. Only 70 people were included in the study. While the model had a high ability to distinguish PAS, the model of placenta accreta, placenta increta, and placenta percreta showed no significant differences. Delli Pizzi et al. (2019) used a 5-point Likert scale scoring method to score eight MRI signs of PAS and established a purely radiological scoring model in 2019, which had a good prediction effect for PI and clinical adverse pregnancy outcomes. However, MRI scoring in PAS did not consider the relationship between placenta previa and previous cesarean incision in PPP, and only 38 patients were included. The AUC of Delli Pizzi’s predictive model for PAS was 0.833, inferior to what we found in our study (0.9197).

Numerous studies have shown that a history of cesarean section and the position of placenta previa are the most important risk factors for PPP (D'Antonio and Bhide, 2014; Jauniaux et al., 2021). In the analysis of the risk etiology of PPP published by Hou et al. (2020), it was found that completely placenta previa (OR = 3.607) and cesarean section more than 2 times (OR = 3.048) were two of the independent risk factors for PPP with PAS, and the hysterectomy rate was also increased. Our PPP scoring scale is intended to directly guide obstetricians to evaluate the possibility of PPP with PAS, so as to fully prepare before surgery and reduce bleeding complications. Table 2 also shows that there are statistical differences in cesarean section history and placenta previa type for PPP patients with PAS of different grades. The scoring scale established by MRI signs alone is slightly less effective than the evaluation of imaging sings and frequency of cesarean section (Supplementary 1). Therefore, based on the risk score of PAS, this scoring scale for PPP included these two risk factors.

In addition, T2 signal signs are important in placental MRI. The normal placental uterine interface has three layers: the medial T2 low-signal decidua layer, intermediate T2 medium-signal muscle layer, and lateral T2 low-signal serosal layer (Novis et al., 2021). A clear T2 low-signal serous layer can be seen on the surface of the normal bladder. Disruption of the normal structure of the uterine wall was observed when the placenta was implanted, and blurring or disruption of the T2 hyposignal serosal layer on the bladder surface was observed if the penetrating placenta had invaded the bladder. Placental MRI for patients with PPP in the middle and late stages of pregnancy can overcome the lack of a clear structural display due to the thin uterine wall before delivery. According to Goergen et al.'s study Goergen et al. (2018), the T2 low-signal band shadow in the placenta is related to fibrin deposition caused by hemorrhage and infarction secondary to PI. The deeper the PI, the lower the signal shadow and the larger its area. Derman et al. (2011) research showed that the deeper the placenta is implanted, the more blood is needed, and the larger and thicker the blood vessels in the placenta. The placental bed is composed of the decidua at the placental uterine interface and its adjacent myometrium. Therefore, the increase in the blood supply required by the placenta of pregnant women with PPP may lead to an increase in the abnormal distribution of blood vessels in the placental bed (Chantraine et al., 2012).

Heterogeneous intraplacental signs can be caused by placental bleeding, infarction, or fibrin deposition. Placental MRI in the middle and late stages of pregnancy may not show uneven signals owing to placental degeneration before delivery. Although this sign is greatly influenced by the gestational age and subjective opinion of the doctor, if it is found in the area of suspected PI, it is still of great significance for diagnosis (Kapoor et al., 2021). Wibke Blaicher’s report Blaicher et al. (2006) indicates that normal placentas of 36–41 weeks often develop a lack of uniformity due to placental maturation and degeneration (Kilcoyne et al., 2017), which may account for a low specificity. In our study, all patients in four groups were subjected to MRI examination at 28–39 weeks of gestation and median gestational age at delivery were 36–37 weeks. Early gestational age with poor placental degeneration can avoid the error of reading for heterogeneous intraplacental sings.

Cervical and bladder invasions are important features of PPP associated with PAS. Because of the low position of the cervix, cervical invasion and formation of surrounding new blood vessels caused by PI are often closely related to pelvic floor blood vessels, resulting in difficult clinical treatment and intraoperative hemorrhage. Ueno et al. (2014) reported MRI signs of placental invasion of the cervix. In their study, the MRI signs were divided into two scoring items: placental position and blurry interruption of the bladder wall, because in patients with PPP, placental placement is usually at the site of the cesarean section scar. PI can cause a large number of new blood vessels to proliferate at the uterine bladder interface. From an anatomical perspective, the vascular lumen is large, wall is thin, and blood flow is rich, showing the characteristics of arterial venalization (de la Chica et al., 2015). If the operation is not carefully managed, bleeding from the surface of the uterus and bladder can still cause massive bleeding. The American College of Obstetricians and Gynecologists 2017 guidelines for postpartum bleeding suggest that blood transfusion should be initiated immediately when a woman has blood loss ≥1500 ml (Bulletin No and Practice, 2017). In this study, when the score was >15.5, the sensitivity and specificity for predicting PI were 0.577 and 0.958, respectively, and the median postpartum blood loss was 1500 ml. Adequate preparation of blood products, collaboration of multidisciplinary teams, and hysterectomy should be considered during preoperative preparation.

Owing to the limited number of samples included in the present study, the results have some limitations, and the predictive efficacy shown herein needs to be verified by prospective studies. Because the grouping was based on clinical and pathological diagnosis, the final distribution of patients into groups was not equalized, which reduces the statistical power. In addition, the prediction efficiency of different MRI signs for PPP combined with PI is different. Therefore, our future goals are to establish a prediction model, conduct external verification using test samples, and increase the sample size.

## Conclusion

In conclusion, the proposed MRI scoring system is an effective diagnostic tool for assessing PPP types and predicting the associated bleeding risk. In the future, the efficacy and extensibility of the MRI scoring model of PPP should be further verified and improved to standardize the application of MRI in the preoperative diagnosis of PPP.

## Data Availability

The raw data supporting the conclusion of this article will be made available by the authors, without undue reservation.

## Abbreviations

PPP, pernicious placenta previa; PI, placental implantation; FIGO, International Federation of Gynecology and Obstetrics; PAS, placenta accreta spectrum disorders; PA, placental adhesion; PP, placenta percreta; vs., versus; US, ultrasonography; MRI, magnetic resonance imaging; T2WI, T2-weighted imaging; TR, repetition time; TE, echo time; ROC, receiver operating characteristic.
