# Supplementary material for: Effectiveness of a fetal magnetic resonance imaging scoring system for predicting the prognosis of pernicious placenta previa: A retrospective study
Source: Front Physiol. 2022 Aug 11;13:921273. doi: 10.3389/fphys.2022.921273 (PMC9402898; doi:10.3389/fphys.2022.921273)
Supplement: Supplementary file 1 [file Table1.docx]

| Supplementary 1. Pathological predictions in the four groups of patients only with MRI image signs. | | | |
| --- | --- | --- | --- |
|  | Compare A | Compare B | Compare C |
| AUC(95% CI) | 0.9135(0.8524-0.9746) | 0.8775(0.8284-0.9266) | 0.856(0.7785-0.9324) |
| Boundary value | 4.5 | 10.5 | 13.5 |
| Specificity | 0.700 | 0.870 | 0.922 |
| Sensitivity | 0.980 | 0.779 | 0.615 |
| Positive predict value | 0.947 | 0.827 | 0.552 |
| Negative predict value | 0.875 | 0.830 | 0.939 |
| Youden index | 0.682 | 0.648 | 0.538 |
| Positive likelihood ratio | 3.267 | 5.992 | 7.885 |
| Negative likelihood ratio | 0.029 | 0.254 | 0.418 |
| Kappa value | 0.75 | 0.65 | 0.51 |

Compare A: Non-placenta accreta vs placenta accreta/placenta increta/placenta percreta；

Compare B: Non-placenta accreta/placenta accreta vs placenta increta/placenta percreta;

Compare C: Non-placenta accreta/placenta accreta/placenta increta vs placenta percreta
